# Optimising non‐cycloplegic screening strategies for early detection of pre‐myopia and myopia in young children

**DOI:** 10.1111/opo.13525

**Published:** 2025-05-14

**Authors:** Síofra Harrington, Michael Moore, James Loughman, Ian Flitcroft, Veronica O'Dwyer

**Affiliations:** ^1^ School of Physics, Clinical & Optometric Sciences Technological University Dublin Dublin Ireland; ^2^ Centre for Eye Research Ireland, Sustainability & Health Research Centre Technological University Dublin, City Campus Dublin Ireland; ^3^ Department of Ophthalmology Mater Misericordiae Hospital Dublin Ireland

**Keywords:** axial length monitoring, myopia, non‐cycloplegic screening, pre‐myopia detection, visual acuity

## Abstract

**Purpose:**

Early detection of myopia is essential to delay its onset and progression. Pre‐myopia, defined by an inadequate hyperopic reserve, increases myopia risk in childhood. However, effective screening methods remain limited. This study aimed to develop practical non‐cycloplegic screening methods for pre‐myopia and myopia in 6‐ to 7‐year‐olds to support earlier interventions.

**Methods:**

This cross‐sectional study of 621 Irish schoolchildren (mean age: 7.12 ± 0.45 years; 51.8% boys) assessed uncorrected distance visual acuity (UDVA). Cycloplegic spherical equivalent refraction (SER) classified refractive status (myopia: SER ≤ −0.50D; pre‐myopia: SER > −0.50 ≤ 0.75D). Pre‐ and post‐cycloplegic SER were measured using the Welch Allyn Spot Vision Screener and Dong‐Yang Rekto‐ORK 11, respectively. Axial length (AL) and corneal radius (CR) were measured with the Zeiss IOLMaster and parental myopia history via questionnaire. Logistic regression and ROC curves evaluated non‐cycloplegic screening methods.

**Results:**

Pre‐myopia prevalence was 24.3% (95% confidence intervals (CI): 29.3–36.2), and myopia prevalence was 3.3% (CI: 2.5–5.5). UDVA screening had an area under the curve (AUC) (CI) = 0.72 (0.59–0.86) and 0.42 (0.36–0.47) for detecting myopia and pre‐myopia, respectively. For pre‐myopia discrimination, non‐cycloplegic SER, AL, AL/CR and parental myopia had AUCs of 0.67 (0.62–0.72), 0.67 (0.62–0.72), 0.69 (0.64–0.74) and 0.59 (0.53–0.64), respectively. The best method combined non‐cycloplegic SER and AL/CR (AUC = 0.72 (0.67–0.76)). Including UDVA or parental myopia did not improve results.

For myopia detection, AUCs were non‐cycloplegic SER:0.84 (0.72–0.97), AL:0.88 (0.82–0.95), AL/CR:0.84 (0.75–0.94) and parental myopia:0.62 (0.48–0.75). The best method combined AL and non‐cycloplegic SER 0.94 (0.90–0.99). Adding parental myopia did not improve the AUC = 0.93 (0.87–0.99) but adding UDVA achieved an AUC = 0.95 (0.90–0.99).

**Conclusion:**

While UDVA alone provided acceptable discrimination for myopia, it was insufficient for screening pre‐myopia. Non‐cycloplegic SER alone had relatively poor discrimination for pre‐myopia, but its performance improved when combined with the AL/CR ratio. The best results for myopia discrimination were achieved by combining non‐cycloplegic SER, axial length and UDVA measures.

**Key points**

- Current school vision screening, designed to detect amblyopia, typically checks only distance visual acuity using a basic threshold and is ineffective for identifying children at risk of developing myopia.
- Cycloplegia is considered essential for detecting early myopia and pre myopia, but limits school screening feasibility; combining axial length and non‐cycloplegic refractive measures offers a practical, accurate and scalable alternative.
- A two‐step approach, with non‐cycloplegic biometric prescreening followed by detailed assessment of high‐risk cases, will support early detection, facilitating timely preventive care for children at risk of myopia progression.

## INTRODUCTION

Myopia has become a significant global public health issue, affecting approximately 30% of the population in 2020, with projections suggesting this will rise to 50% by 2050.^1^ The irreversible complications of high myopia, such as retinal maculopathy and glaucoma,^2^ highlight the importance of early detection and prevention. Current interventions, ranging from increased outdoor activities^3^ to treatments such as atropine,^4^ specialist myopia control spectacle lenses,^5^, ^6^ soft bifocal contact lenses^7^ and orthokeratology,^8^ have demonstrated efficacy in slowing myopia progression and delaying myopia onset.^9^ Therefore, early identification and timely intervention are essential for managing myopia effectively in children.

In recent years, the age of myopia onset has shifted to younger populations.^10^ For instance, among Chinese children, the average age of onset decreased from 12 years in 2010 to 10 years in 2014, and further to 7 years of age in 2019.^11^ Recognising this trend, the International Myopia Institute (IMI) introduced the concept of pre‐myopia in 2019.^12^ Defined as a refractive state with a spherical equivalent refraction (SER) ≤ +0.75D and >−0.50D at 6 years of age, and ≤0.50D at ages 7–8 years, with specific risk factors and growth patterns significantly increasing the likelihood of developing myopia.^12^ Despite this, most research on myopia prediction has focused on school‐aged children in Asia,^13^, ^14^, ^15^, ^16^ highlighting the need for screening models tailored to other regions and populations.

While pre‐myopia is recognised as a public health concern in Asia,^14^ it is also a significant issue in predominantly white European populations. For example, longitudinal research in Northern Ireland found that children aged 6–7 years with <+0.75D SER, or a cut‐off of +0.63D, were likely to develop myopia by age 13.^17^ In contrast, children who remained emmetropic at 13 years of age had, on average, 1.07D of hyperopia at 6–7 years.^17^ These findings emphasise the importance of identifying pre‐myopia.^12^ Additionally, the most rapid level of myopia progression appears to occur in the early phase of onset, with axial elongation preceding myopia onset and the onset of symptoms.^18^, ^19^ Hence, predicting which children are likely to experience this acceleration in eye growth and progression to myopia and detecting those who have already started the accelerated eye growth phase but have not yet become myopic is critical as both are candidates for preventive treatment.

Vision screening is vital in identifying children at higher risk of developing health conditions, enabling timely interventions to reduce incidence and complications.^20^ Hence, screening for pre‐myopia offers a critical opportunity to monitor children with an insufficient hyperopic reserve, implement interventions to delay or prevent the onset of myopia and mitigate its long‐term risks. Current vision screening for schoolchildren often relies on uncorrected distance visual acuity (UDVA).^21^ In Ireland, vision screening for reduced visual acuity (0.20 logMAR or worse in one or both eyes) is typically performed around 6 years of age or during the first year of school.^22^ However, studies have shown that UDVA has a high false‐positive rate (33.6%), a low positive predictive value (29.5%–51.1%)^23^ and a limited predictive value for detecting pre‐myopia.^15^

Similarly, non‐cycloplegic autorefraction, though widely used in population‐based studies, tends to underestimate hyperopia and overestimate the prevalence of myopia^24^ and pre‐myopia in children.^25^ While cycloplegic refraction is the gold standard for detecting refractive errors,^26^ its routine use in school screenings is limited due to concerns over eye drop administration and potential side effects.^27^

Given the limitations of current methods, there is a pressing need to develop accurate, non‐cycloplegic screening models for detecting pre‐myopia and myopia in younger children. This cross‐sectional study focused on 6‐ to 7‐year‐old schoolchildren in Ireland, assessing visual acuity (VA), non‐cycloplegic photo screening autorefraction and ocular biometrics—specifically axial length (AL) and corneal radius of curvature (CR)—to create effective and practical non‐cycloplegic screening models for pre‐myopia and myopia.

## METHODS

The study's sampling, recruitment protocols, participation rates and experimental methods have been detailed previously.^28^ Stratified random sampling was employed to obtain a representative sample of children from mainstream schools in Ireland, with stratification based on urban or rural residence and socioeconomic status.

Ethical approval was granted by the Technological University Dublin Research Ethics Committee and the study adhered to the principles of the Declaration of Helsinki. Before data collection, written informed consent was obtained from all participants' parents/legal guardians. Child assent was obtained on the day of testing.

Data collection occurred between June 2016 and January 2018, involving 621 schoolchildren (377 boys) from Ireland. Ethnic representation included 559 White participants and 62 non‐White participants (27 Black, 14 East Asian, 11 South Asian and 6 Arab). The child's biological parent's myopia history was self‐reported by the parent or legal guardian.

Children whose parents or legal guardians provided written informed consent and the child's assent were examined at their school during school hours. The examination included the measurement of monocular uncorrected distance logMAR visual acuity (UDVA) using the Good‐Lite Sloan chart (store.good‐lite.com) at 3 m. Visual acuity was recorded using a by‐letter scoring system and expressed in logMAR notation. A light meter was used to ensure the test luminance remained at or above 120 cd/m^2^.

Non‐cycloplegic autorefraction was measured by photo screening using the Spot Vision Screener (Spot; Welch Allyn Inc., welchallyn.com). The Spot Vision Screener has been described in prior studies.^29^ In brief, the examiner selected the appropriate age range on the device's home screen. The Spot was approximately 1 m from the participant (the device advises moving closer to or further away from the subject). The device's twinkling lights and sounds served as fixation targets for participants. Measurements were generally taken binocularly and took about 2 s; for children with strabismus, measurements were taken monocularly.

Axial length (five measurements) and corneal radius (three measurements) were obtained using the Zeiss IOLMaster 500 (Carl Zeiss, Meditec Inc., zeiss.com). The mean corneal radius was calculated as the average of the steepest and flattest corneal radii. The axial length/corneal radius ratio (AL/CR) was defined as the AL divided by the mean CR.

Refractive error was determined using cycloplegic autorefraction using a Dong Yang Rekto ORK 11 Auto Ref‐Keratometer (Everview Corp., everview.co.kr). The eye drop protocol used in this study was consistent with previous epidemiological studies involving similar‐aged children.^30^, ^31^, ^32^ To achieve sufficient cycloplegia while minimising systemic side effects, one drop of topical anaesthetic (Minims proxymetacaine hydrochloride 0.5% w/v, Bausch.com) was administered, followed by one drop of 1% cyclopentolate hydrochloride (Minims 1% w/v, Bausch.com) for participants with blue, green or grey irides. Brown or hazel irides received two drops of cyclopentolate hydrochloride, instilled 5 min apart. If the pupillary light reflex remained present after 20 minutes, a third drop was instilled.^30^, ^31^, ^32^

Cycloplegia was confirmed at least 20 min after eye drop administration by assessing pupillary non‐responsiveness to light and ensuring an accommodative amplitude of <2D using the push‐up test. Once cycloplegia was established, autorefraction was performed. SER, calculated as the sphere plus half the cylindrical value, was used for analysis.

Follow‐up: After the examination, all parents/legal guardians received a detailed report of the study findings and the need for any further treatment if required.

### Statistical analyses

Ocular biometric, refractive and UDVA data for participants' right and left eyes were highly correlated; thus, data analysis was conducted using measurements from the right eye only.^33^ Logistic regression models were employed to develop joint screening methods, incorporating UDVA, non‐cycloplegic SER and ocular biometry to identify myopia and pre‐myopia.

Baseline characteristics were summarised as counts and proportions for categorical variables and mean ± standard deviation (SD) for continuous variables. Chi‐squared tests were used to compare categorical variables; logistic regression was used to assess the relationship between pre‐myopia, myopia and socioeconomic and demographic variables, while the Kolmogorov–Smirnov test assessed the normality of variable distributions. For normally distributed variables, differences between two groups were analysed using Student's *t*‐test, and differences among the three groups were evaluated using one‐way analysis of variance. For non‐normally distributed variables, the Wilcoxon rank‐sum test was applied.

Receiver operating characteristic (ROC) curves were constructed for each model to determine the optimal cut‐off values, the area under the curve (AUC) and Youden's indices. Cycloplegic SER was used as the reference standard for the ROC analyses, with myopia defined as SER ≤ −0.50D and pre‐myopia defined as SER ≤ +0.75D and >−0.50D. Non‐myopia was classified as a SER > +0.75D following cycloplegia. For the ROC analysis for myopia discernment, the control group consisted of children classified as non‐myopic, based on cycloplegic refraction. This included children with SER > −0.50D. For pre‐myopia, a two‐step approach was adopted where myopia was first identified, followed by pre‐myopia screening among non‐myopic children. Sensitivity and specificity were also calculated to evaluate model performance. Statistical analyses were conducted using IBM SPSS Statistics (version 29.0, ibm.com) and RStudio (version 2023.09.1 + 494, r‐project.org).

## RESULTS

The mean age of the participants was 7.12 years (SD = 0.45, range: 6.01–7.99 years). There was no significant difference in mean age between boys (7.10 (0.47) years) and girls (7.13 (0.46) years), *p* = 0.39.

Table 1 displays the study characteristics. There was no significant difference in the prevalence of myopia between boys and girls. Parental myopia was associated with pre‐myopia (Odds ratio = 2.6 (95% confidence intervals (CI): 1.33–5.09, *p* = 0.005)) but not myopia (*p* = 0.12).

**TABLE 1:** General characteristics and summary statistics of study variables of the 621 participants between 6 and 7 years of age.

	Overall	SER > 0.75D	SER > −0.50 & ≤0.75D	SER ≤ −0.50D	Odds ratio (95% CI)^a^	*p*‐Value
Prevalence, *n* (%), [95% CI]	621 (100)	450 (72.5) [68.7–75.9]	151 (24.3) [21.0–27.9]	20 (3.2) [2.0–5.0]		
Age (mean ± SD)	7.12 (0.45)	7.06 (0.46)	7.24 (0.44)	7.41 (0.43)		
Sex						
Male, *n* (%)	322 (51.9), [47.8–55.8]	233 (72.4)	77 (23.9)	12 (3.7)	0.99 (0.70–1.4)	0.95
Female	299 (48.2), [44.2–52.2]	217 (72.6)	74 (24.7)	8 (2.7)		
Ethnicity						
White, *n* (%)	559 (90.0), [87.3–92.2]	409 (73.2)	138 (24.7)	12 (2.1)	1.40 (0.80–2.44)	0.24
Non‐white	62 (10.0), [78.0–12.7]	41 (66.1)	13 (21.0)	8 (12.9)		
Living environment						
Rural *n* (%)	354 (57.0), [53.0–60.9]	243 (68.9)	97 (27.4)	14 (4.0)	1.58 (1.09–2.27)	0.02
Urban, *n* (%)	267 (43.0), [39.08–47.0]	207 (77.5)	54 (20.2)	6 (2.2)		
Socioeconomic status						
Disadvantaged, *n* (%)	178 (28.7), [25.2–32.4]	148 (83.1)	23 (12.9)	7 (3.9)	2.30 (1.48–3.58)	<0.001
Advantaged, *n* (%)	443 (71.3), [67.6–74.8]	302 (68.2)	128 (28.9)	13 (2.9)		
Parental myopia						
Neither parent	338 (54.4)	263 (77.8)	70 (20.7)	5 (1.5)	Ref	
One myopic parent	203 (32.7)	134 (66.0)	60 (29.6)	9 (4.4)	1.46 (0.74–2.87)	0.28
Both parents	42 (6.8)	24 (57.1)	17 (50.5)	1 (2.4)	2.63 (1.36–5.10)	0.004
Missing^b^	38 (6.1)					
VA (logMAR, mean ± SD)	0.042 (0.215)	0.045 (0.213)	0.002 (0.193)	0.271 (0.280)		<0.001
AL (mean ± SD)	22.53 (0.80)	22.39 (0.76)	22.83 (0.74)	23.62 (0.22)		<0.001
AL/CR (mean ± SD)	2.89 (0.09)	2.87 (0.09)	2.93 (0.07)	3.02 (0.11)		<0.001
Non‐cycloplegic photo screener SER (mean ± SD)	0.51 (0.97)	0.66 (1.06)	0.23 (0.29)	−0.70 (0.86)		<0.001
Cycloplegic SER (mean ± SD)	1.46 (1.29)	1.91 (1.19)	0.47 (0.27)	−1.12 (0.95)		<0.001

Abbreviations: AL, axial length; AL/CR, axial length/ mean corneal radius ratio; CI, 95% confidence intervals; D, dioptre; max, maximum; min, minimum; mm, millimetres; N, n, Number; SD, standard deviation; SER, spherical equivalent refraction; VA, visual acuity.

^a^ Odds of being myopic (≤−0.50D) or pre‐myopic (>−0.50 to ≤0.75) with spherical equivalent refraction >0.75D as reference category.

^b^ Parental myopia relates to participants' birth parents; some legal guardians did not have this information.

As anticipated, UDVA was worse in myopes compared to pre‐myopes and non‐myopes. Axial length was longest in myopes, followed by pre‐myopes and shortest in non‐myopes. Similarly, the AL/CR ratio was highest in myopes, followed by pre‐myopes and non‐myopes. Additionally, non‐cycloplegic SER measured with the Welch Allyn Spot Vision Screener was more myopic in myopes than pre‐myopes, who were more myopic than the non‐myopes (Table 1).

Figure S1 illustrates scatter plots showing the relationships between the quantitative screening tools and cycloplegic SER used in the study. The relationship between UDVA and cycloplegic SER was weak (*R* ^2^ = 0.03), nonlinear and closer to being quadratic (*R* ^2^ = 0.19). In contrast, stronger correlations were observed between cycloplegic SER and non‐cycloplegic SER (*R* ^2^ = 0.51), as well as with AL (*R* ^2^ = 0.23) and the AL/CR ratio (*R* ^2^ = 0.34).

**FIGURE 1:**
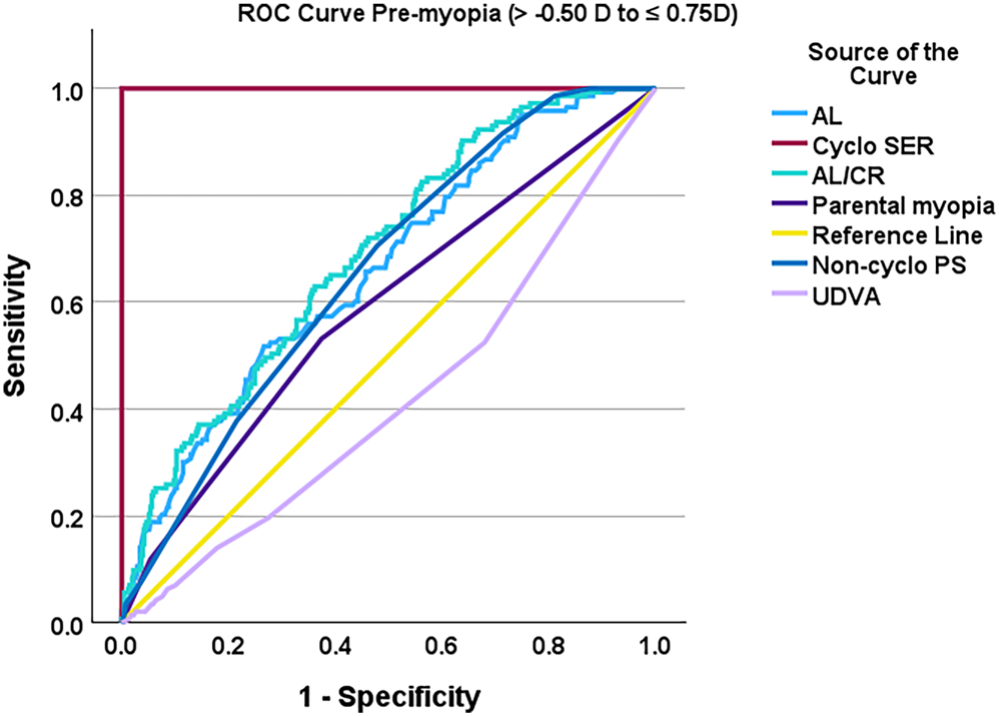
Receiver operating characteristic (ROC) curves for the individual screening tests for pre‐myopia (>−0.50 to ≤0.75D) with cycloplegic spherical equivalent refraction (Cyclo SER) as the reference standard (red line). Axial length (AL), axial length/corneal radius ratio (AL/CR), non‐cycloplegic photo screening (Non‐cyclo PS), uncorrected distance visual acuity (UDVA).

**FIGURE 2:**
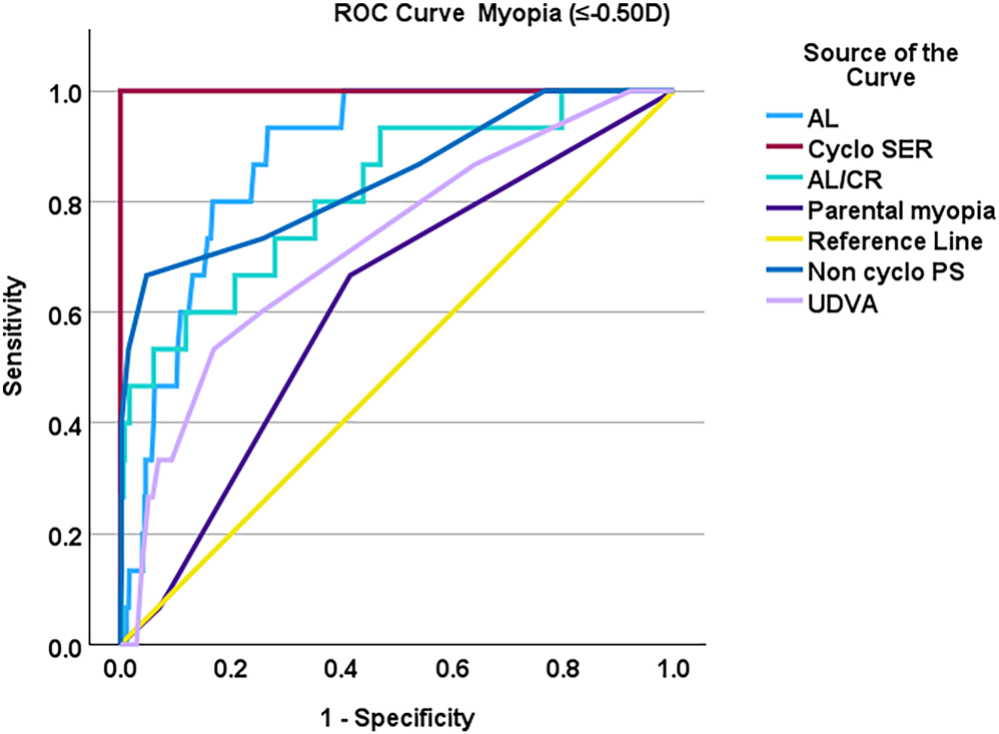
Receiver operating characteristic (ROC) curves for the individual screening tests for myopia (≤−0.50D) with cycloplegic spherical equivalent refraction (Cyclo SER) as the reference standard (red line). Axial length (AL), axial length/corneal radius ratio (AL/CR), non‐cycloplegic photo screening (Non‐cyclo PS), uncorrected distance visual acuity (UDVA).

**FIGURE 3:**
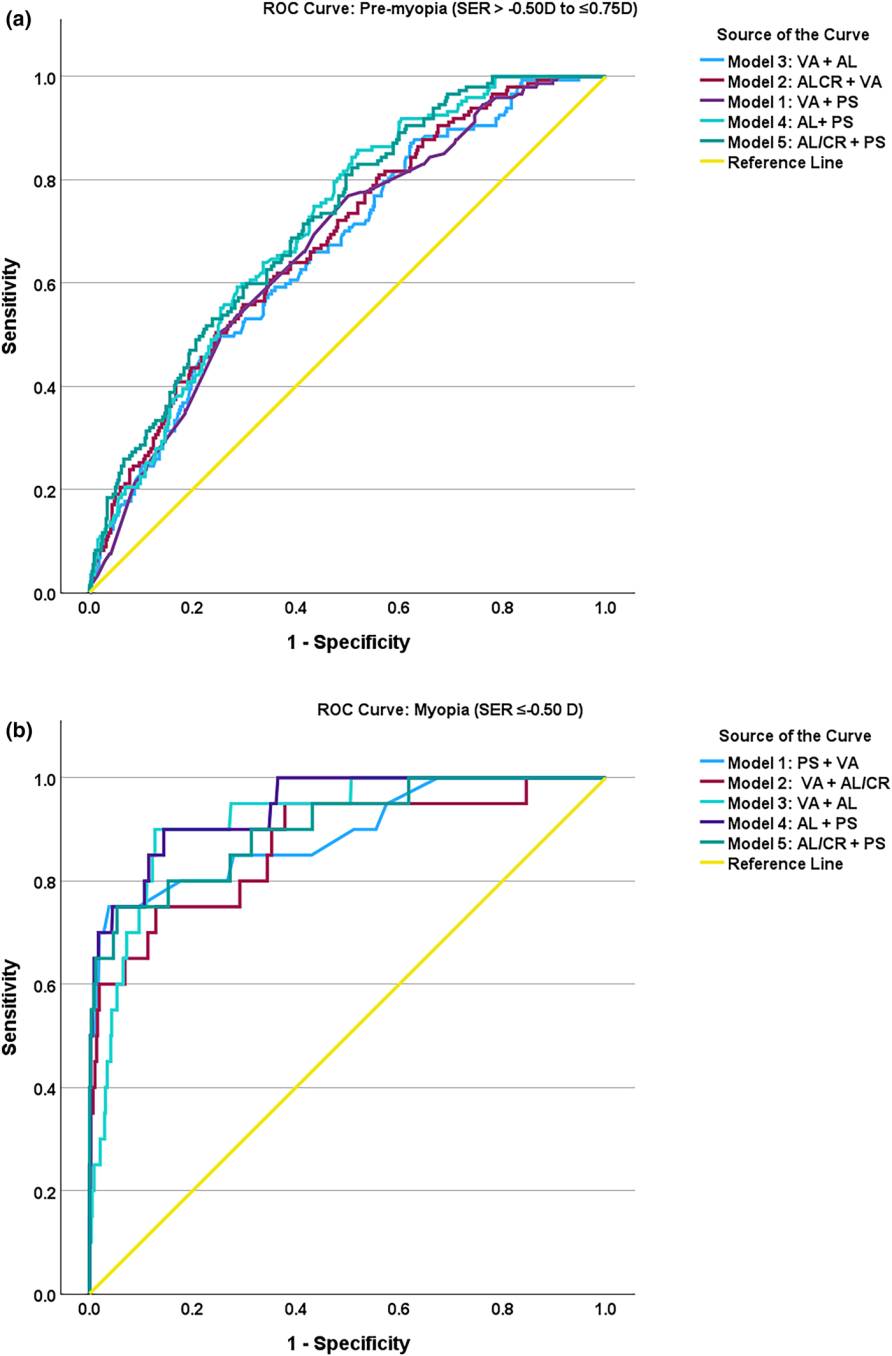
Discriminative performance for the combined screening models for pre‐myopia (>−0.50 to ≤0.75) (plot a) and myopia (≤−0.50) (plot b). Uncorrected distance visual acuity (VA), axial length (AL), axial length/corneal radius ratio (AL/CR), non‐cycloplegic spherical equivalent refraction photo screening (PS), receiver operating characteristic curves (ROC).

Figure 1 displays the ROC curves for the individual screening tools, while Table 2 presents the screening discriminative performance of the various methods for detecting myopia and pre‐myopia The findings indicate that discriminative performance was high for AL (AUC = 0.88, 95% CI: 0.82–0.94), AL/CR (AUC = 0.84, 95% CI: 0.75–0.94) and non‐cycloplegic (SER 0.84, 95% CI: 0.72–0.97), which demonstrated strong discriminative performance for myopia screening. However, for pre‐myopia screening, their discriminative performance was fair (AUC ranging from 0.64 to 0.68) (refer to Table 2). For pre‐myopia, the AL/CR ratio had the highest AUC (0.69, 95% CI: 0.64–0.74; Figures 2 and 3).

**TABLE 2:** Summary of single‐indicator discriminative performance screening for myopia and pre‐myopia.

Measurement	Cut‐off value^a^	Sensitivity^a^	Specificity^a^	AUC^b^
	Screening target: Myopia (SER ≤ −0.50D, post instillation of cycloplegic)			
Visual acuity	0.150	0.533	0.832	0.72 (0.59–0.86)
Axial length	22.965	0.933	0.734	0.88 (0.82–0.94)
AL/CR	2.974	0.667	0.793	0.84 (0.75–0.94)
Photo screener (non‐cycloplegic)	−0.125	0.733	0.743	0.84 (0.72–0.97)
Parental history		0.667	0.586	0.62 (0.48–0.75)
	Screening target: pre‐myopia (SER > −0.50 and ≤0.75D, post cycloplegia)			
Visual acuity	0.05	0.196	0.726	0.42 (0.36–0.47)
Axial length	22.825	0.517	0.733	0.67 (0.62–0.72)
AL/CR	2.905	0.629	0.628	0.69 (0.64–0.74)
Photo screener (non‐cycloplegic)	−0.375	0.706	0.521	0.67 (0.62–0.71)
Parental history		0.531	0.626	0.59 (0.53–0.64)

Abbreviations: AL, axial length; AUC, area under the curve; CR, corneal radius of curvature; SER, spherical equivalent refraction.

^a^ Accuracy when the Youden's index was maximised.

^b^ Mean (95% confidence interval).

Table 3 shows the discriminative performance of combined screening tools incorporating two instruments. The best combination for myopia combined AL with non‐cycloplegic SER (AUC = 0.94, 95% CI: 0.90–0.99) and pre‐myopia AL/CR with non‐cycloplegic SER (AUC = 0.72, 95% CI: 0.67–0.76). Adding UDVA as a third parameter improved the performance for myopia (AUC = 0.95, 95% CI: 0.90–0.99) but did not improve the accuracy for pre‐myopia (AUC = 0.71, 95% CI: 0.66–0.75).

**TABLE 3:** Accuracy for combined screening models for myopia and pre‐myopia in 621 schoolchildren aged 6–7 years in Ireland.

Model	Measurement				Myopia						Pre‐myopia					
	VA	AL	AL/CR	PS	Sen		Spec		YI	AUC (95% CI)	Sen		Spec		YI	AUC (95% CI)
1	√			√	0.75		0.96		0.71	0.89 (0.80–0.98)	0.77		0.49		0.26	0.67 (0.62–0.72)
2	√		√		0.75		0.87		0.62	0.87 (0.78–0.96)	0.64		0.64		0.26	0.69 (0.64–0.74)
3	√	√			0.90		0.87		0.77	0.92 (0.87–0.97)	0.49		0.75		0.24	0.67 (0.62–0.72)
4		√		√	0.90		0.86		0.76	0.94 (0.90–0.99)	0.85		0.51		0.33	0.71 (0.67–0.76)
5			√	√	0.90		0.69		0.70	0.90 (0.83–0.98)	0.83		0.49		0.32	0.72 (0.67–0.76)

*Note*: Uncorrected visual acuity (VA), axial length (AL), axial length/mean corneal radius ratio (AL/CR), non‐cycloplegic photo screener (PS), sensitivity (Sen), specificity (Spec), Youden's index (YI), area under the curve (AUC), confidence intervals (CI). 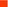, Excellent ≥ 90%; 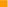, Good 80%–89%; 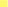, Fair 70%–79%; 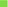, Poor 60%–69%; 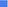, Not clinically useful < 60%.

Including parental myopia in the models did not improve their performance for myopia (AUC = 0.93, 95% CI: 0.87–0.99) or pre‐myopia (AUC = 0.73, 95% CI: 0.69–0.78).

## DISCUSSION

This study evaluated the effectiveness of various non‐cycloplegic screening methods for detecting myopia and pre‐myopia in 6‐ to 7‐year‐old children in Ireland, contributing to ongoing efforts to refine school‐based screening strategies. While UDVA demonstrated moderate discriminative performance for myopia (53%), it was poor for pre‐myopia (20%), reinforcing its limited value as a standalone screening tool, mainly due to its low sensitivity to early refractive changes associated with pre‐myopia. Among the screening methods evaluated, the combination of non‐cycloplegic SER (measured with the Welch Allyn Photo Screener) and AL demonstrated the highest overall performance for myopia detection (sensitivity: 90%, specificity: 86%) and AL/CR for pre‐myopia detection (sensitivity: 84%, specificity: 49%), achieving a balance between sensitivity and specificity. For myopia detection, this approach yielded a high area under the curve (AUC = 0.94), reflecting strong discriminative ability, making it a viable alternative to conventional VA screening.

The inclusion of UDVA or parental myopia history in the models did not improve screening accuracy significantly (myopia sensitivity: 87.5%, specificity: 85%; pre‐myopia sensitivity: 80%, specificity: 52%), suggesting that these factors may be less reliable predictors in early myopia detection frameworks. While previous research demonstrates that self‐reported parental myopia is generally reliable, it is less accurate for hyperopia and astigmatism.^34^ Future research should consider validating parental refractive status to refine risk prediction models.

The findings align with prior research,^15^ highlighting the importance of AL/CR over AL alone when screening for pre‐myopia in young children. The AL/CR ratio accounts for variations in corneal curvature, allowing better differentiation between pre‐myopia and emmetropia in children. By providing a nuanced understanding of axial elongation relative to corneal dimensions, it helps identify subtle ocular growth patterns that might otherwise go unnoticed, making it particularly valuable in distinguishing pre‐myopia from emmetropia. However, pre‐myopia detection accuracy remained lower than for myopia, likely due to stronger accommodation in younger children^35^ and dynamic refractive development^36^ at this age. Despite these challenges, the high prevalence of pre‐myopia in this cohort of 6‐ to 7‐year‐olds (24.3%) highlights the importance of early identification, as one‐third of pre‐myopic children develop myopia within a year, with a 2‐year cumulative risk exceeding 50%.^37^

This study builds on previous work by evaluating the combined predictive value of non‐cycloplegic SER, AL and AL/CR in a European cohort of 6‐ to 7‐year‐old schoolchildren. While non‐cycloplegic screening methods have been widely explored in Asian populations, their applicability in European settings remains uncertain due to lower baseline myopia prevalence^38^ and potentially different refractive development trajectories.^38^ For example, Yin et al.^15^ reported an AUC of 0.74 for pre‐myopia and 0.94 for myopia using non‐cycloplegic SER and AL/CR in preschool‐aged children; figures that compare with the AUCs of 0.72 and 0.90, respectively, in the present study. Similarly, Zhang et al.^39^ and Wang et al.^13^, ^14^ have validated refractive error reference thresholds as predictive markers of myopia onset in non‐myopic Chinese children, highlighting the role of hyperopic reserve as a key risk indicator. Additionally, Liu et al.^16^ found that higher baseline AL and AL/CR were significantly associated with an increased risk of both pre‐myopia and myopia in preschool‐aged cohorts. Although these prior studies focused on younger Asian populations, the current findings extend their relevance by confirming the predictive value of these non‐cycloplegic biometric parameters in a European population, supporting their use in early screening strategies beyond high‐prevalence regions.

The present study also introduces a two‐step screening approach, where myopia is identified first, followed by targeted pre‐myopia screening among non‐myopic children. While Wang et al.^13^ found that combining UDVA with non‐cycloplegic refraction improved myopia detection, the present results demonstrate that incorporating AL/CR enhances pre‐myopia screening performance, although discrimination remains suboptimal. This sequential strategy, not previously evaluated in European cohorts, may improve early detection and screening efficiency.

Early pre‐myopia detection is critical, as timely lifestyle and environmental interventions—such as increased outdoor activity,^40^ reduced near work^41^ and improved visual hygiene^42^—can significantly delay myopia onset^43^, ^44^, ^45^ and reduce long‐term risks like retinal detachment and myopic maculopathy.^2^ With the recent proposal by the United States National Academies of Science, Engineering and Medicine (NASEM) to designate myopia as a disease,^46^, ^47^ the importance of early intervention is highlighted further.

Addressing childhood myopia at a population level also offers the opportunity to mitigate its significant societal and economic impact. Unaddressed myopia and pre‐myopia in childhood have the potential to contribute to increased healthcare costs, diminished productivity and long‐term disability,^48^ while also negatively impacting quality of life and mental well‐being.^49^ Scalable, cost‐effective public health strategies, including awareness campaigns, school‐based education and broader access to affordable myopia control treatments^50^ will be key to reducing the long‐term burden of myopia and improving lifelong visual and social outcomes.

This study provides a practical framework for improving myopia and pre‐myopia detection in school‐based vision programmes by refining non‐cycloplegic screening methods and exploring a two‐step screening strategy. These findings contribute to developing population‐specific screening guidelines and highlight the need for further validation across diverse cohorts. A key strength is the demonstration that combining non‐cycloplegic SER with AL yields high discriminative accuracy for myopia detection in young children. This approach achieved a sensitivity of 90%, specificity of 86% and an AUC of 0.94, providing a robust alternative to traditional tools such as UDVA, which showed poor accuracy for pre‐myopia detection.

However, several limitations should be considered. The combined use of non‐cycloplegic photorefraction with AL/CR for identifying pre‐myopia was suboptimal (AUC = 0.72), despite composite indices. This level of discernment was comparable to that of UDVA screening for myopia (AUC = 0.72), the current standard for in‐school screening in Ireland. This may reflect the physiological overlap between pre‐myopic and emmetropic refractive states,^51^ making differentiation more difficult, particularly in the absence of cycloplegia. Additionally, axial elongation in pre‐myopes may not yet be sufficiently distinct at this young age,^52^ further reducing diagnostic accuracy.

The study also relied on high‐cost equipment, including the IOLMaster and Welch Allyn Spot Vision Screener, which may limit the scalability of these methods in resource‐constrained settings, particularly when compared with the lower cost of VA screening. Furthermore, the study was conducted in Ireland, so the findings may not be fully generalisable to populations with different demographic or environmental profiles.

Future research should focus on evaluating emerging portable ocular biometers assessing their cost‐effectiveness and feasibility for large‐scale pre‐myopia screening in diverse populations. Considering the importance of delaying myopia onset through environmental and behavioural interventions, the long‐term public health benefits of early pre‐myopia identification will be invaluable.^53^ At an individual level, early detection may contribute to better visual outcomes and improved quality of life.^54^, ^55^

## CONCLUSION

This study provides a comprehensive evaluation of screening techniques for myopia and pre‐myopia in 6‐ to 7‐year‐old schoolchildren in Ireland, emphasising the importance of ocular biometric measurements. Findings indicate that UDVA alone lacks sufficient accuracy, while AL alone demonstrated superior discriminative performance for myopia detection. However, none of the individual methods detected pre‐myopia reliably.

Under non‐cycloplegic conditions, combining AL/CR with SER improved screening effectiveness, although pre‐myopia detection remains challenging. A two‐step screening strategy presents a novel and promising approach to enhancing early identification, particularly in school‐based, non‐cycloplegic programmes. Expanding public health initiatives and refining screening methodologies will be essential to reducing the long‐term impact of myopia and protecting visual health for future generations.

## AUTHOR CONTRIBUTIONS

**Síofra Harrington:** Conceptualization (lead); data curation (lead); formal analysis (lead); funding acquisition (lead); investigation (lead); methodology (lead); project administration (lead); resources (equal); software (equal); supervision (equal); validation (equal); visualization (lead); writing – original draft (lead); writing – review and editing (lead). **Michael Moore:** Conceptualization (supporting); data curation (supporting); formal analysis (supporting); investigation (supporting); methodology (supporting); project administration (supporting); resources (equal); software (equal); validation (supporting); visualization (supporting); writing – original draft (supporting); writing – review and editing (supporting). **James Loughman:** Conceptualization (supporting); data curation (supporting); formal analysis (supporting); funding acquisition (supporting); investigation (supporting); methodology (supporting); project administration (supporting); resources (supporting); software (equal); supervision (supporting); validation (supporting); visualization (supporting); writing – original draft (supporting); writing – review and editing (supporting). **Ian Flitcroft:** Conceptualization (supporting); data curation (supporting); formal analysis (supporting); funding acquisition (supporting); investigation (supporting); methodology (supporting); project administration (supporting); resources (supporting); software (equal); supervision (supporting); validation (supporting); visualization (supporting); writing – original draft (supporting); writing – review and editing (supporting). **Veronica O'Dwyer:** Conceptualization (supporting); data curation (supporting); formal analysis (supporting); funding acquisition (equal); investigation (supporting); methodology (supporting); project administration (equal); resources (equal); software (supporting); supervision (lead); validation (supporting); visualization (supporting); writing – original draft (supporting); writing – review and editing (supporting).

## FUNDING INFORMATION

This work was supported by the Technological University Dublin Fiosraigh Scholarship and additional funding from Optometry Ireland. The funding organisations had no role in the design or conduct of this research.

## CONFLICT OF INTEREST STATEMENT

No conflicting relationship exists for any author.

## PATIENT CONSENT STATEMENT

Before data collection, written informed consent was obtained from all participants' parents/legal guardians. Child assent was obtained on the day of testing.

## Supporting information


Figure S1.

## Data Availability

No additional data are available.
