# Supplementary figures and images for: Optimising non‐cycloplegic screening strategies for early detection of pre‐myopia and myopia in young children
Source: Ophthalmic Physiol Opt. 2025 May 14;45(5):1080–9. doi: 10.1111/opo.13525 (PMC12153036; doi:10.1111/opo.13525)

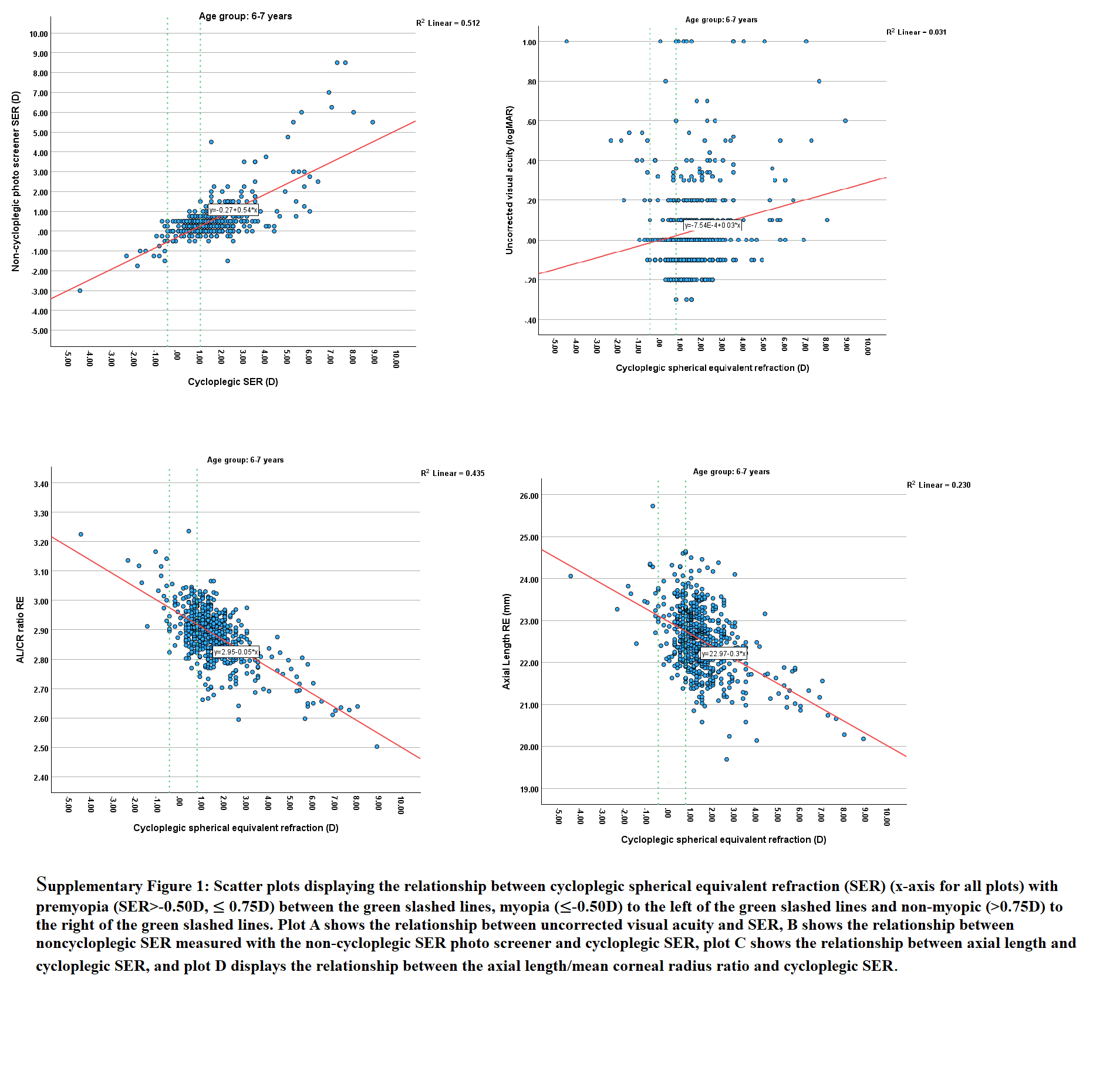

Supplement: Supplementary file 1 — Figure S1. [file OPO-45-1080-s001.tif]
